# Evaluation of raw material extraction, processing, construction and disposal of cement and concrete products: datasets and calculations

**DOI:** 10.1016/j.dib.2019.103929

**Published:** 2019-04-18

**Authors:** David A.C. Manning, Napaporn Tangtinthai, Oliver Heidrich

**Affiliations:** a School of Natural and Environmental Sciences, Newcastle University, Newcastle upon Tyne, NE1 7RU, UK; b Waste and Hazardous Substance Management Division, Pollution Control Department, Ministry of Natural Resources and Environment, 92 Soi Phahonyothin 7, Phahonyothin Rd., Phayathai, Bangkok 10400 Thailand; c School of Engineering, Newcastle University, Newcastle upon Tyne, NE1 7RU, United Kingdom

## Abstract

To evaluate the material flows associated with construction and demolition in different countries it is necessary to have a consistent set of data. However, data collected by regulators and governments differ and this study used concrete as a case in point. Concrete is a significant man-made material in construction whose use reflects socio-economic variation between countries. Flows of natural components, cement and aggregates, are investigated from extraction to final disposal following demolition (Tangtinthai et al., 2019). The housing sector dominates the use of concrete in urbanized areas and greatly reflects socio-economic and resource extraction issues. To compare concrete stock, use and policies of contrasting countries the data from Thailand and Great Britain (GB) are considered, but as reported they differ for each country. We present here the results of the calculations required to generate an internally consistent database for Great Britain and for Thailand that enables an informed materials flow analysis to be undertaken on materials consumed and generated during construction and demolition of concrete structures. The research methodology and calculations for national cement and concrete production (including clinker, cement kiln dust, gypsum, and aggregates) and the resulting datasets help to make projections that shape policy requirements for Thailand and other emerging economies as reported in (Tangtinthai et al., 2019).

**Table:** Specifications table

Subject area	Civil engineering, mining
More specific subject area	Resource management, waste management, MFA
Type of data	Material quantities
How data was acquired	Calculations based on stoichiometry and other physical-chemical relationships
Data format	Tables and calculations to quantify material flow analysis (MFA)
Experimental factors	Data taken from published sources were processed to provide an internally consistent set of data for MFA
Experimental features	No experimental work was carried out; the calculations use published data
Data source location	The data used relate to production statistics for cement and concrete. Data sources include UK (e.g. Office of National Statistics, Mineral Products Association, London), Thailand (e.g. National Statistical Office, Pollution Control Department, Bangkok) government websites, and international industry websites.
Data accessibility	All the data that form the basis for the calculations are openly accessibly or are with this article
Related research article	Tangtinthai, N., O. Heidrich, and D.A.C. Manning, Role of policy in managing mined resources for construction in Europe and emerging economies. Journal of Environmental Management, 2019. 236, 613–621 [1]

**Table:** 

**Value of the data** • This Data in Brief provides a consistent set of data for material flow associated with cement and concrete. • The approach taken here has wide geographical application and enables policy formulation to be based on evidence. • The data facilitate policy development relating to waste and resource management in countries with rapid urban growth.

## 1 Data

The data presented here are calculated for use in material flow analysis.

First, the raw material inputs for cement manufacture are calculated for both the UK and Thailand, based on the stoichiometry of the calcining reaction (Table 1). The raw materials required to produce clinker are given in Table 2. Corresponding quantities of raw materials for UK and Thai clinker production are given in Table 3. Cement production requires fuel, in amounts that depend on the process used (Table 4). Finally, overall production, imports and exports of clinker are summarised in Table 5.

**Table 1:** Loss of volatiles during calcining.

Raw Materials	Limestone	Shale
Proportion	75%	25%
Chemical components	CaCO_3_	MgO, SiO_2_, Al_2_O_3_, CaO, Fe_2_O_3_, etc
Weight loss in firing	44%^a^	10%^b^
Weight after firing	56%	90%

a Based on decomposition reaction [4]: CaCO_3_ = CaO + CO_2_.

b Based on typical loss of CO_2_ + H_2_O from shale [4].

**Table 2:** Raw material required for production of clinker, tonnes.

Raw Materials (t)	Limestone	Shale
Weight	75	25
Weight after firing	42	22.5
Amount of raw material needed per tonne of clinker	1.16^a^	0.39^b^

a Calculated as 75/(42 + 22.5).

b calculated as 25/(42 + 22.5).

**Table 3:** Raw material requirements, based on production of clinker and by-product cement kiln dust (CKD).

Raw materials (Mt)	Great Britain	Thailand
Clinker production	6.56	39.55
CKD production	0.44^a^	2.64^a^
Limestone	8.12	48.94
Shale	2.73	16.45
Total	10.85	65.39

a Based on production of 6.67 t CKD by-product per 100 t clinker [5].

**Table 4:** Fuel use.

	Great Britain		Thailand
Assumptions [8]	22% semi dry/semi wet	78% dry	100% dry
Specific heat consumption	5 MJ/kg clinker	3.2 MJ/kg	3.2 MJ/kg
Conventional fuels (coal)	60%	60%	60%
Alternative fuels	40%	40%	40%
Calorific value of conventional fuels (coal) [9]	28.30 MJ/kg	28.30 MJ/kg	28.30 MJ/kg
Calorific value of alternative fuels [9]	18.20 MJ/kg	18.20 MJ/kg	18.20 MJ/kg
Combined calorific value	24.26 MJ/kg	24.26 MJ/kg	24.26 MJ/kg
Fuel requirement (kg/kg clinker)^a^	0.21	0.13	0.13
Total fuel requirement^b^	0.32 Mt	0.71	5.48
Conventional fuel requirement	0.19 Mt	0.43	3.29
Unconventional fuel requirement [10]	0.13 Mt	0.28	2.19
Total conventional fuel consumption	0.62 Mt		3.29
Total alternative fuel consumption	0.41 Mt		2.19
Fuel ash [9]	0.1 Mt		0.55 Mt
CO_2_ emissions	4.88		28.68

a Based on specific heat consumption.

b Based on proportion of clinker produced in each process.

**Table 5:** Overall clinker production, imports and exports.

Mt	Great Britain [11]	Thailand [7]
Manufactured clinker	6.56	39.55
Imported clinker	0.21	0.37
Exported Clinker	0.03	7.22^a^
Net domestic clinker	6.74	32.70

a Includes 6.19 Mt clinker for cement manufacture and 1.03 Mt clinker for other uses [4].

Secondly, inputs required to make concrete are provided by either using data from referenced sources, or calculations based on technical proportions. Table 6 gives estimates of cement quantities, which then feed into use for mortar (Table 7) and for concrete (Table 8).

**Table 6:** Material data for cement production.

Mt	Great Britain [11]	Thailand [7]
Net domestic clinker	6.74	32.70
Gypsum^a^	0.35	1.72
Cement imports	1.46	0.20
Other additives [6]	1.60	7.54^b^
Exported to make cement	0.31	7.00
Exported for other purposes	0.21	1.03
Total cement for domestic use	9.63	33.95

a 5% cement.

b Assumes same proportion as Great Britain.

**Table 7:** Requirements for mortar production.

Mt	Great Britain	Thailand
Total cement for domestic use	9.63	33.95
Cement used for mortar	2.20 [14]	3.88^a^
Sand used for mortar	5.47 [15]	9.65^b^
Lime for mortar	0.63	1.11
Total mortar	8.30	14.64

a Overall, it is assumed that 5% of cement is used for purposes such as soil and pH stabilisation [12]; the corresponding figure for Great Britain is 3% [13]. This study uses the Great Britain proportion and assumes the same proportion to estimate total mortar, halved to reflect different building practices. 1) Great Britain uses brick and block as a double masonry layer with an inside cavity [16], 2) Great Britain used 46% brick and 41% concrete & mortar by weight for residential building [17], 3) The main Thai construction materials are concrete (79.4% by weight) followed by 13% brick and 5.6% steel respectively [18]. 4) Thai C&D waste is 74.9–79.4% concrete by weight [19].

b Assumes same proportion of fine aggregate to Great Britain.

**Table 8:** Material use for concrete.

Mt	Great Britain	Thailand
Cement	7.43	30.07
Fine aggregates [20]	19.70	79.73
Coarse aggregates [20]	28.34	134.93
Recycled and secondary aggregates [1]	5.00	0.00
Sub-total	60.47	244.73
Additives (excluding water; 4.5% of total) [21]	2.85	11.53
Total	63.32	256.26
Waste during delivery (0.5%)	0.32	1.28
Lime for mortar (3.5 t cement requires 1 t lime)	0.63	1.11
Waste during construction (5%)	3.57	13.48 [19]

Finally, quantities of concrete stocks in housing and other construction types are calculated (Table 9).

**Table 9:** Proportions of construction and concrete use (2012).

Buildings	Great Britain [22], [23]		Thailand [18], [24]	
	Percent	Mt	Percent	Mt
Residential building	37.01	25.07	51.51	131.94
*Including* single-residential building	25.17	17.05	41.90	107.32
*Including* multi-residential building	11.84	8.02	9.61	24.62
Non-residential building	44.07	29.85	32.19	82.45
Infrastructure	18.92	12.81	16.30	41.75
Total	100.00	67.73	100.00	256.14

## 2 Research methodologies, datasets and calculations

We utilize knowledge of the chemical reactions involved in calcination [4] to estimate amounts, where statistical data are lacking, of raw materials used in the clinker and cement manufacturing processes. Data for the year 2012 for national cement production in Great Britain (data are available for Great Britain, not the United Kingdom, which consists of Great Britain and Northern Ireland) and Thailand are combined with quantities for coarse and fine aggregate use, assuming similar mixing ratios for Great Britain and Thailand. Data for components such as fuels (conventional and alternative) and cement kiln dust (CKD), are derived from official statistics and publications from the global cement industry and annual recorded clinker production. The results are summarised in Table 1, Table 2, Table 3

CO_2_ emissions are calculated based on weight loss after mineral calcination and fuel combustion (Table 4). Clinker quantities are used to calculate the equivalent amounts of cementitious products for import and export (Table 5). Table 6 summarises material quantities for cement trade for each country; Table 7, Table 8 give quantities for mortar and concrete used in construction. We calculate [1] the annual concrete flows for different construction sectors: single-residential building, multi-residential building, non-residential building and infrastructure, whose proportions are summarised in Table 9.

A brief comparison of national datasets such as population, economy, and urbanisation, building lifespan, policy and regulation [2], [3] is described [1]. The calculations presented lead to recommendations and environmental taxes that are adapted from the EU and Great Britain and their impact, if implemented, on ASEAN countries is described [1].
